# Assessment of Pharmacogenomic Panel Assay for Prediction of Taxane Toxicities: Preliminary Results

**DOI:** 10.3389/fphar.2017.00797

**Published:** 2017-11-07

**Authors:** Raffaele Di Francia, Luigi Atripaldi, Salvo Di Martino, Carla Fierro, Tommaso Muto, Anna Crispo, Sabrina Rossetti, Gaetano Facchini, Massimiliano Berretta

**Affiliations:** ^1^Hematology-Oncology Unit, Istituto Nazionale Tumori, Fondazione “G. Pascale” IRCCS, Napoli, Italy; ^2^Hematology and Cellular Immunology (Clinical Biochemistry), A.O. dei Colli Monaldi Hospital, Naples, Italy; ^3^CETAC Research Center, Caserta, Italy; ^4^Epidemiology-Oncology Unit, Istituto Nazionale Tumori, Fondazione “G. Pascale” IRCCS, Napoli, Italy; ^5^Medical Oncology Unit, Istituto Nazionale Tumori, Fondazione “G. Pascale”, Napoli, Italy; ^6^Department of Medical Oncology, CRO National Cancer Institute, Aviano, Italy

**Keywords:** genotyping methods, *ABCB1*, *ABCG2*, CYP3A4^*^1B, CYP2C8^*^3, *ERCC2*, *XRCC3*

## Abstract

**Backbone:** Paclitaxel and docetaxel are the primary taxane anticancer drugs regularly used to treat, breast, gastric, ovarian, head/neck, lung, and genitourinary neoplasm. Suspension of taxane treatments compromising patient benefits is more frequently caused by peripheral neuropathy and allergy, than to tumor progression. Several strategies for preventing toxicity have been investigated so far. Recently, findings on the genetic variants associated with toxicity and resistance to taxane-based chemotherapy have been reported.

**Methods:** An extensive panel of five polymorphisms on four candidate genes (ABCB1, CYP2C8^*^3, CYP3A4^*^1B, XRCC3), previously validated as significant markers related to paclitaxel and Docetaxel toxicity, are analyzed and discussed. We genotyped 76 cancer patients, and 35 of them received paclitaxel or docetaxel-based therapy. What is more, an early outline evaluation of the genotyping costs and benefit was assessed.

**Results:** Out of 35 patients treated with a taxane, six (17.1%) had adverse neuropathy events. Pharmacogenomics analysis showed no correlation between candidate gene polymorphisms and toxicity, except for the *XRCC3* AG+GG allele [OR 2.61 (95% CI: 0.91–7.61)] which showed a weak significant trend of risk of neurotoxicities vs. the AG allele [OR 1.52 (95% CI: 0.51–4.91)] *P* = 0.03.

**Summary:** Based on our experimental results and data from the literature, we propose a useful and low-cost genotyping panel assay for the prevention of toxicity in patients undergoing taxane-based therapy. With the individual pharmacogenomics profile, clinicians will have additional information to plan the better treatment for their patients to minimize toxicity and maximize benefits, including determining cost-effectiveness for national healthcare sustainability.

## Introduction

Numerous physicians evidence the significance of genetics variants in drug response and suggest the use of information's genetic testing to plan personalized treatment. In practice, pharmacogenetic testing can stratify patients who are less likely to benefit from expensive treatments and those who develop toxicities at standard doses. This enables both more tailored alternative treatments and perhaps a reduction in delays for patients. For these reasons, pharmacogenomic and pharmacogenetic (PGx) tests are attractive in the field of anti-cancer drugs.

The toxicity profiles of paclitaxel and docetaxel are well-documented; toxic reactions to these taxane-based drugs often lead to a reductionin benefits for patients and the discontinuation of treatment. Mainly acute peripheral neuropathy has been linked to acute and cumulative doses of taxane in terms of toxicities (Scripture et al., 2006).

Mechanisms of neurotoxicity are related to microtubule disturbances in the dorsal root ganglia, axons, and Schwann cells. Many efforts have been made in an attempt to develop strategies for reducing toxicities (e.g., using neuroprotective agents), although these attempts have yielded only modest achievement (Di Francia et al., 2013). Moreover, to a large extent, inter-individual variability in neurotoxicity remains unexplained. In the last decade, numerous PGx studies have reported several single nucleotide polymorphisms (SNPs) associated with the same adverse drug response in cancer (De Monaco et al., 2014). These have shown that neurotoxicity related to the taxane treatment can be predicted through the identifications of gene polymorphisms known to be involved with taxane transports, biotransformation, and DNA damage repair gene (Frederiks et al., 2015; Boora et al., 2016).

Recently, the well-known synonymous SNP *ATP-binding cassette subfamily B member 1* (*ABCB1 alias MDR1*) *3435 C*>*T* (rs1045642) showed a notably lower overall survival rate than the CC genotype for the allele variant, in patients with metastatic breast cancer (Kus et al., 2016). Another study found greater clearance of docetaxel in patients with the Cytochrome P450 (CYP) 3A4^*^1B and CYP3A5^*^1A alleles (Baker et al., 2009). Other small studies have found lower clearance of paclitaxel related to the CYP2C8^*^3 allotype (Gréen et al., 2009).

The DNA repair protein *X-Ray Cross-Complementing group 3* (*XRCC3*) is an element of the double-strand break repair machinery. Its diminished activity is related to a drastically elevated grade of DNA breaks and theoretically to higher efficacy of anticancer agents. *XRCC3* Thr241Met *316A*>*G* (rs1799794) polymorphism is associated with severe non-hematological toxicity (Qiu et al., 2013). Numerous studies have shown this relation to be statistically important, but lots of others have failed to do so (Tran et al., 2006; Hertz et al., 2014).

Based on this scientific evidence, we have validated a genotyping panel assay containing the most relevant pharmacogenomic markers, including, ***ABCB1** (Alias MDR1)*, CYP3A4^*^1B, CYP2C8^*^3, and ***XRCC3***. Additional SNPs on *Glutathione S-Transferase* 1 ***GSTP1*** Ile105Val, *Excision-Repair Cross-Complementing group 2 (**ERCC2** alias XPD)* Lys751Val, CYP3A4^*^22, Solute Carrier Organic 1B1 (**SLCO1B**1) Val174Ala, *and ATP-binding cassette subfamily G member 2 (**ABCG2**)*. Val12Met were evaluated.

The assessment of these SNPs should provide valuable predictive results on both acquired and heritable adverse reactions in patients treated with a taxane. However, most of these findings should be validated in larger clinical trials (Marsh et al., 2007).

If the detection and predictive value of these SNPs on aforecited genes are regularly incorporated into clinical procedures, the personalized therapy should be scheduled (Di Francia et al., 2012a). However, an accurate evaluation of usefulness of the PGx tests, in terms of comparative costs and benefits is still ongoing. Currently, the literature is weak in terms of policy and trials exploring the pharmacoeconomic impact of a genetic test in patients who receive taxanes. However, the choice of genotyping platforms is fundamental in terms of cost-effectiveness studies on PGx (Di Francia et al., 2010). A pertinent model is provided by the National Institute for Health and Clinical Excellence (NICE). NICE constitutes a Diagnostic Advisory committee that aims to encourage Biotech-Pharma and academic communities to promote comprehensive sets of data, in economic models of healthcare (Dhalla et al., 2009).

The goal of this experimental pilot work is to establish a validated genotyping panel assay for the prevention of neurotoxicity in patients for whom taxane-based therapy is planned.

Oncologists will thus have a new tool aimed at both toxicity and/or to adopting the optimal scheduling approach with a view to minimizing cumulative neurotoxicity in taxane-based therapy.

## Materials and methods

### Patient selection

The samples were collected to the National Institute “and CETAC Research Center Policy” of Naples, Italy. This retrospective work was performed in compliance with the ethical values laid down by the Declaration of Helsinki, and informed consent documentation was reviewed and agreed by the independent ethics committee and CETAC Research Center policy. The study was planned to measure whether the PGx profile can affect taxane-induced neurotoxicity. In total, 76 cancer patients (29 male and 47 female) were enrolled, 35 of whom received adjuvant taxane chemotherapy (Table 1).

**Table 1:** Distribution of selected variables according to Taxane users (*n* = 35) vs. no taxane (Control cohort *n* = 41): Univariate analysis.

	**Patients**		*p*-value^*^	OR (95% CI)^**^
	**Control cohort *n* (%)**	**Taxane users *n* (%)**		
Age			0.06	
<60	17 (41.5)	22 (62.9)		1
≥60	24 (58.5)	13 (37.1)		0.42 (0.16–1.06)
Gender			0.04	
Male	20 (48.8)	9 (25.7)		1
Female	21 (51.2)	26 (74.3)		2.75 (1.04–7.29)
Type of cancer			nd	
Breast	14	7		
Genitouranary	8	11		
Gastric	1	2		
Other	18	15		
Adverse events			**0.001**	
No	29 (70.7)	12 (34.3)		1
Yes	12 (29.3)	23 (65.7)		4.7 (1.82–12.6)
Neutro & Neuro			**0.003**	
No	29 (70.7)	12 (34.3)		1
G1 & G2	11 (26.8)	18 (51.4)		4.09 (1.49–11.18)
G3 & G4	1 (2.4)	5 (14.3)		12.5 (1.32–118.47)
Neutropenia			**0.02**	
No	37 (90.2)	24 (68.6)		1
Yes	4 (9.8)	11 (31.4)		4.35 (1.24–15.25)
Neuropathy			0.3	
No	37 (90.2)	29 (82.9)		1
Yes	4 (9.8)	6 (17.1)		1.96 (0.51–7.62)

* Chi-Square test;

** *Crude odds ratio logistic regression were adjusted for age and gender. In bold are significative results*.

All patients had a diagnosis of carcinoma (primarily, breast, ovarian, genitourinary, etc.) and were treated with paclitaxel- or docetaxel-based therapy. The chemotherapy dose, schedule, and duration were as follows: for paclitaxel 175 mg/m^2^ intravenously (IV) every 3 weeks for 4 cycles, (for adjuvant treatment for breast cancer) and/or 80 mg/m^2^ weekly IV for 12 cycles, and for docetaxel IV 100 mg/m^2^ for 4 cycles for firstline metastatic cancer.

The sample included 39 patients aged <60 years (51.3%) and 37 aged ≥60 years, who were separately analyzed and evaluated in relation to risk factors for neurotoxicity. The patients were separated into two arms: those with and those without neurotoxicity. Grade <2 and grade ≥2 neurotoxicity were also individually registered.

The inclusion criteria were: patients >18 years old, male and female, Eastern Cooperative Oncology Group performance status of 0–1, histologically proven cancer, and on a paclitaxel or docetaxel regimen. All enrolled patients were without comorbidity causative of peripheral neuropathy (i.e., diabetes). At the time of the neurotoxicity event, the patients were not receiving other anticancer agents.

### Assessment of neurotoxicity

The assessment of neurotoxicity was evaluated based on symptom narration, the presence of symmetrical “stocking-glove” numbness, loss of deep tendon reflexes, and burning and/or tingling after therapy. Baseline taxane-induced peripheral neurotoxicity was assessed according to the National Cancer Institute Common Toxicity Criteria for Adverse Events (NCI-CTCAV) version 4.0 grading scale from 0 to 4:

0 = normal;
1 = asymptomatic, weakness on physical examination, loss of reflexes, or paresthesias not interfering with daily functioning;
2 = weakness and sensory alterations interfering with daily functioning;
3 = weakness and sensory changes interfering with activities of daily living or requiring bracing or assistive devices;
4 = life-threatening, paralysis, disabling.

### Pharmacogenetic assay

Genomic DNA was extracted with a mouth swab in accordance with the manufacturer's protocol using an Ampli-DNA extraction kit (Dia-Chem, srl, Naples, Italy).

The genotyping assay was performed using the TaqMan probe-based chemistry allelic discrimination assay in the OneStep platform (Life Technologies, Monza, Italy). The investigating panel test included the CYP2C8^*^3, CYP3A4^*^22, GSTP1, ERCC2, SLCO1B1, ABCG2, and *XRCC3* polymorphisms. The reaction mix and temperature protocol (95°C for 15″ and 60°C for 1 min for 40 cycles) were performed in accordance with the manufacturer's protocol (Life Technologies, Monza, Italy). The primers and probe were designed by PrimerExpress 3.0 (Life Technologies) for the allelic discrimination assays (Supplementary Table 1). In addition, *MDR1* and CYP3A4^*^1B assay were performed using Ampli-MDR1 and Ampli-CYP3A4 kits (Dia-Chem, srl) in order to confirm the previously reported data (Bosch et al., 2006; Kus et al., 2016).

### Statistical analysis

Differences according to age, gender, and adverse events, in particular for neutropenia and neuropathy, between taxane users and the control cohort were calculated using the Chi-square test. Univariate analyses were performed to match the two arms: the unadjusted logistic regression method was used to assess crude odds ratios (ORs) and 95% confidence intervals (CIs). Logistic regression models adjusted for major confounders like age and gender were used to calculate adjusted ORs and 95% CIs for each gene variants risk factors. Analyses were performed using SPSS for Windows, version 23.0 (IBM Corporation, NY, USA). A bilateral *p* < 0.05 was considered statistically significant.

## Results

### Patient reports

Thirty-five cancer patients (26 female and 9 male) who received adjuvant taxane therapy were enrolled in this retrospective study. Of these, 23 (65.7%), had experienced an adverse event, and 5 (14.3%) of them were >grade 2 (Table 1). Six (17.1%) patients experienced neurotoxicity. In the sample, 22 subjects were under 60 years old, and 13 were over the age of 60 years. The sharing of genetic SNPs in agreement with risk factors is listed in Table 2. The control cohort was represented by 41 cases who did not receive a taxane; they were treated primarily with platinum and fluoropyrimidine-based chemotherapy. It is worth noting, is that the case cohort (taxane users) recorded more adverse events than the control cohort, with 23 (65.7%) and 12 (28.6%), respectively (*p* = 0.001). In addition, more taxane users experienced toxicity than the control, with 5 (14.3%) and 1 (2.4%), respectively (*p* = 0.003).

**Table 2:** Distribution of genetic polymorphism according to risk factors (any grade of neutropenia and neuropathy).

**Gene variants**	**Patients**		*p*-value^*^	OR (95% CI)^**^
	**Control cohort *N* = 41 (%)**	**Taxane users *N* = 35 (%)**		
ABCB1 Iso1145Iso			0.50	
“CC”	15 (36.6)	13 (37.1)		1
“CT”	23 (56.1)	18 (51.4)		0.57 (0.20–1.66)
“TT”	3 (7.3)	4 (11.5)		1.67 (0.26–10.67)
ABCB1 Ala893Ser			0.40	
“GG”	15 (36.6)	19 (54.3)		1
“GT/A”	22 (53.7)	14 (39.6)		0.56 (0.21–1.47)
“TT/AA”	4 (9.8)	2 (6.1)		0.44 (0.07–2.76)
CYP3A4^*^1B 5′UTR			0.70	
“AA”	29 (70.7)	26 (74.3)		1
“AG+GG”	12 (29.3)	9 (25.7)		0.60 (0.20–1.83)
CYP2C8^*^3 Lys399Arg			0.19	
“TT”	28 (75.7)	25 (71.4)		1
“CT”	6 (16.2)	10 (28.6)		1.62 (0.49–5.35)
“CC”	3 (8.1)	0		n.d.
CYP3A4^*^22 intron 6			0.30	
“CC”	38 (92.7)	30 (85.7)		1
“CT”	3 (7.3)	5 (14.3)		2.17 (0.48–9.79)
GSTP1 Iso105Val			0.70	
“AA”	25 (70.0)	22 (62.9)		1
“AG”	16 (30.0)	13 (37.1)		1.25 (0.44–3.60)
SLCO1B1 Val174Ala			0.60	
“TT”	27 (65.8)	27 (77.1)		1
“CT”	12 (29.3)	8 (22.9)		1.03 (0.35–3.06)
“CC”	2 (4.9)	0		n.d.
ABCG2 Val12Met			0.40	
“CC”	35 (85.4)	27 (77.1)		1
“CT”	4 (9.8)	3 (8.6)		1.0 (0.2–4.84)
“TT”	2 (4.8)	5 (14.3)		3.33 (0.6–18.5)
ERCC2 Lys751Gln			0.40	
“TT”	22 (53.7)	14 (40)		1
“GT”	14 (34.1)	15 (42.9)		1.76 (0.66–4.71)
“GG”	5 (12.2)	6 (17.1)		1.97 (0.51–7.68)
XRCC3 Thr241Met			**0.03**	
“AA”	34 (82.9)	23 (65.7)		1
“AG”	7 (17.1)	7 (20.0)		1.52 (0.5–4.91)
“GG”	0	5 (14.3)		n.d.
“AG”+“GG”		12 (34.3)		2.61 (0.90–7.61)

* Chi-Square test;

** *Crude odds ratio logistic regression were adjusted for age and gender. In bold are significative results*.

*Distribution of genetic polymorphism according to neutropenia and neurotoxity with the distinction for grading among 35 Taxane users only, are provided in the Supplementary Table 2*.

### Genotyping assay

Several criteria were considered for selecting gene variants for the pharmacogenomic panel tests: (i) search on the whole standardized polymorphisms acknowledged to influence the pharmacokinetics/pharmacodynamics of taxanes (www.pharmgkb.org); (ii) review of current researches, particularly trials including polymorphisms related to toxicity; (iii) identification of issues related to the impact of genotyping testing which might provide answers concerning the incorporation of PGx markers in clinical practice.

The genotypes of all genes analyzed in this study and their relation with any grade of neutropenia and neuropathy are summarized in Table 2. Distribution of genetic polymorphism according to neutropenia and neurotoxity, with the distinction for grading among 35 Taxane users only are provided (Supplementary Table 1).

The *ABCB1 3435C*>*T* rs1045642 genotype of taxane users was divided into two groups: TT allele vs. CT+CC alleles (4 cases, 11.5%). The OR for every toxicity grade was 1.67 (0.26–10.67, *P* = 0.50), when compared with CT+CC (medium and low risk, respectively) allele genotype.

The *ABCB1 2677G*>*T/A* rs2032582 genotype was divided into two groups: TT/AA allele vs. GT/A and GG alleles (2 cases, 6.10%). OR for every toxicity grade was 0.44 (95% CI: 0.07–2.76, *P* = 0.40), when compared with GT/A+ GG alleles (medium and low risk, respectively) allele genotype.

The CYP3A4^*^1B−*392A*>*G* rs2740574 genotype was divided into two groups: AG+GG risk allele vs. AA alleles (9 cases, 25.7%). OR for every toxicity grade was 0.60 (95% CI: 0.20–1.83, *P* = 0.70), when compared with AA (low risk) allele genotype.

CYP2C8^*^3 Lys399Arg rs10509681 genotype was divided into two groups: CT allele vs. TT alleles (10 cases, 28.6%). OR for every neuropathy grade was 1.62 (95% CI: 0.49–5.35, *P* = 0.19), when compare with CT (medium risk) allele genotype.

The CYP3A4^*^22 intron 6 rs35599367 genotype was divided into two groups: CC allele vs. CT genotypes, No homozygous for TT were detected. The OR for any neuropathy grade was 2.17 (95% CI: 0.48–9.79, *P* = 0.30), in a boon of the CT genotype.

The *GSTP1* Iso105Val rs1695 genotype was divided into two groups: GG allele vs. AG+AA genotypes. The OR for any grade neuropathy was 1.25 (95% CI: 0.44–3.60, *P* = 0.71).

The *ERCC2 2251T*>*G* Lys751Gln rs13181 *(alias XPD)* genotype was divided into three groups: TT vs. GT and GG genotypes. The OR for any grade neuropathy was 1.76 (95% CI: 0.66–4.71, *P* = 0.41), and the OR for GG was 1.97 (95% CI: 0.51–7.68, *P* = 0.41).

The *SLCO1B1* Val174Ala rs4149056 genotypes was were separated into two groups: TT vs. CC+CT genotypes. The OR for any grade neuropathy was 1.03 (95% CI: 0.35–3.06, *P* = 0.62).

The *ABCG2 G34A* Val12Met rs2231137 genotype was divided into three groups: GG vs. GA and AA genotypes. The OR for any neuropathic grade was 1.00 (95% CI: 0.20–4.84, *P* = 0.41), for GA, and the OR for AA was 3.33 (95% CI: 0.61–18.5, *P* = 0.41).

The *XRCC3 316A*>*G* Thr241Met rs1799794 genotype was divided into two groups: AA vs. GA and GG genotypes. The OR for any neuropathy grading was 1.52 (95% CI: 0.51–4.91) for GA alleles, and the OR for GG+GA was 2.61 (95% CI: 0.91–7.61) *P* = 0.03.

### Genotyping costs

Multiple genotyping methods have been validated for assessing the mutational profile of the mentioned SNPs, but no gold standard has been defined. Moreover, only a few studies have addressed the cost-effectiveness of pharmacogenomic testing in terms of the implications for clinical practice (Payne and Shabaruddin, 2010; Tirelli et al., 2011). For instance, Van den Akker-van Marle et al. (2006) integrated thiopurine S-methyltransferase (TPMT) genotyping prior to scheduling 6-mercaptopurine in pediatric Acute Lymphoblastic Leukemia (ALL); the mean calculated cost has been estimated to average around €150,00 (Van den Akker-van Marle et al., 2006).

In a further report, an early outline of the genotyping costs for “home-made tests” using allele discrimination on the fluorescent-based platform, was calculated at about €20,00 per SNP (Di Francia et al., 2011). Realistic selection of the optimal method in terms of costs per samples is dependent on the specific test provided by laboratories (Di Francia et al., 2010). Our PGx panel assay identifies 5 polymorphisms, and it cost is averaged to €100,00.

Moreover, the major issues to be considered by clinical laboratories providing genotyping services, are: (i) the ease of use of FDA-approved tests; (ii) the lack of government reimbursement; (iii) the need for genotyping accuracy; and (iv) the requirement to find expert clinicians who areable to correctly understand the PGx results (Di Francia et al., 2012b).

## Discussion

Predictive genetic signatures allow oncologists to achieve better cancer therapy. Furthermore, the clinical utility of the selected SNPs implicated in taxane-based therapy is in part restricted by the following (i) the limited diffusion of genotyping in routine diagnostic procedures; (ii) a lack of concrete verification that PGx information improves health; and (iii) the cost-effectiveness of testing is still an open query.

The goal of our study is to propose a validated PGx panel assay for the prevention of neurotoxicity. We developed an inexpensive panel test using the TaqMan “allelic discrimination platform” including the homogeneous detection of five polymorphisms on four genes: ABCB1 (alias MDR1), CYP3A4^*^1B, CYP2C8^*^3, and *XRCC3*. As shown previously, polymorphisms in *ABCB1* and CYP3A4^*^1B, are able to predict taxane neurotoxicity (Kus et al., 2016). The our results for *ABCB1* (alias MDR1) *ABCB1 3435C*>*T* allele TT and CYP3A4^*^1B 392A>*G* AG+GG does not confirm the previously published data due to low cohort of taxane users (Bosch et al., 2006; Kus et al., 2016). Here, we evaluated additional SNPs on the candidate genes: CYP3A4^*^22, GSTP1, ERCC2, SLCO1B1, and *ABCG2*, but did not observe a significant relationship with neutropenia and neurotoxicity except for *XRCC3 316A*>*G* rs1799794 for GG+AG alleles (*p* = 0.03). Despite the low correlation with taxane toxicity (see Results), we believe that any of these polymorphisms could play a key role in the metabolism of taxane (CYP2C8^*^3, CYP3A4^*^22) and the acquired cellular resistance due to DNA repair genes (*ERCC2, XRCC3)*; this is why they were included in the proposed genotyping panel assay (Table 3).

**Table 3:** Knowledge-base of genotype profile of Taxane treatment good/bad responding patients.

**PGx Profile**	**ABCB1 alias MDR1**		CYP2C8^*^3	CYP3A4^*^1B	**XRCC3**	**Effects**
Rs^#^code Nucleotide Codon	rs104564 23435C>T I1145I	rs2032582 2677G>T/A A893S	rs10509681 771 A>G^#^ K399R	rs2740574 −392A>G 5′UTR	rs1799794 316A>G T241M	
MAF^*^	T = 0.566	A = 0.43 T = 0.01	G = 0.131	G = 0.015	A = 0.27	Referred to Caucasian Population
Genotype A	CC	GG	TT	AA	AA	Lower neurotoxicity, due to wild type polymorphisms
Genotype B	CT	GT/A	CT or TT	AG or GG	AG	PM for CYP2C8^*^3, probable toxicity
Genotype C	TT	TT/AA	CT or TT	AG or GG	AG or GG	Very high risk of cumulative Neuropathy caused by high plasma level of taxane due to PM profiles (CYP2C8^*^ and CYP3A4^*^22) and low high extrusion from the cells (ABCB1). In addition, probable acute neutropenia (XRCC3)

* *MAF, Minor Allele Frequency. PM, poor Metabolizer; source: www.ensembl.org/Multi/Search/Results?q=MAF;_site=ensembl;_page=1;_facet_feature_type=Gene*.

# *Design of the primers and probes were made on complementary DNA strand (T > C)*.

*PM, Poor Metabolizer*.

For *ABCB1*, two SNPs (rs1045642 and rs2032586) have been related to the upper serum level of docetaxel, and grade 2–3 neurological toxicity compared to patients with other genotypes (Kim et al., 2015). In particular, grade ≥2 neurotoxicity has been found to be highly recurrent in patients with the *ABCB1 3435TT* allotype in comparison to the CC/TC (OR: 2.76, 95% CI: 1.17–6.49, *P* = 0.017) (Kus et al., 2016). The same study showed that the CYP3A4^*^1B 392AA and AG alleles are predictive of only grade >1 neuropathy, (OR 2.26, 95% CI: 1.03–4.94, *P* = 0.038) (Kus et al., 2016).

Several observational PGx types of research using genome-wide association studies (GWASs) have focused on SNPs related to taxane neurotoxicity, but the results have still been inconclusive and are not sufficiently clinically relevant. The polymorphism CYP2C8^*^3 gene (rs10509681) has been found to be related to a decrease in the metabolic activity of paclitaxel, and associated with potential increases in neuropathy risk (Bergmann et al., 2011). A further study found that breast cancer patients with the CYP2C8^*^3 allele achieved further clinically relevant outcomes using adjuvant paclitaxel (55 vs. 23%; OR: 3.92, 95% CI: 1.46–10.48, corrected *P* = 0.046) but a higher frequency of >grade 2 neurotoxicity was recorded (22 vs. 8%; OR: 3.13, 95% CI: 0.89–11.01, *p* = 0.075). No difference was found in either European-American or African-American patient cohorts (Hertz et al., 2013).

Many studies have found CYP2C8^*^3 to be statistically significant, but many others have failed to do so (Kus et al., 2016), as has our study. We discovered a statistically insignificant relationship between neurotoxicity (6 cases) and polymorphism for the *CYP2C8 CC* genotype (OR: 1.62, 95% CI: 0.49–5.35, *p* = 0.19), compared to the CT (medium-risk) genotype.

Another study of 239 patients receiving paclitaxel, performed the CYP2C8^*^3, CYP2C8^*^4, CYP3A4^*^22, and *ABCB1 3435 C*>*T* genotypes. CYP3A4^*^22 carriers were correlated with an increased risk of severe neuropathy (*P* = 0.043). In addition, this study showed that poor metabolizers (PMs) for CYP3A4^*^22 GG polymorphism were related to severe neurotoxicity of paclitaxel compared to the TT and CT genotypes (De Graan et al., 2013). In our study, the OR was 2.17 (95% CI: 0.48–9.79, *P* = 0.30), in favor of the CT genotype.

In addition, the *GSTP1* Ile105Val polymorphism *313A*>*G* (alias GSTP1^*^B), was related to low enzyme “Glutathione detox” capacity (Mir et al., 2009). As previously demonstrated, in patients with adeno-colorectal cancer treated with a 5-FU and oxaliplatin schedule, the GSTP1 Ile105Val heterozygous status was related with an augmented risk of neuropathy, while patients with Val/Val status had a lower neurotoxicity risk profile and tumor aggressiveness than Ile/Ile phenotypes (Ruzzo et al., 2007). To date, no evidence has been reported for taxane neurotoxicity. This GSTP1313A>G variant may be identified by a simple and cheap allelic discrimination method (Fontana et al., 2009). Given such evidence, we genotyped the taxane users and control cohort but found no statistically relevant correlations.

*SLCO1B1* encodes the organic anion-transporting polypeptide (OATP1B1), whose primary function is the hepatic uptake of a variety of xenobiotics, including taxane (König et al., 2006). A well-known SNP rs4149056 *521 T*>*C*, Val174Ala in the *SLCO1B1* gene is known to lead the transport action of OATP1B1, determining an augmented serum concentrations of numerous drugs (i.e., statin).

To date, there is minimal evidence suggesting an active function for *ABCG2* in paclitaxel transmembrane transporting. Moreover, overexpression of *ABCG2* has been associated with taxane resistance *in vitro* (Brooks et al., 2003).

It is known that the DNA repair system is a principal mechanism for direct (i.e. platinum agent) and indirect (i.e., docetaxel) resistance to chemotherapy. Since the cell is capable of restoring the damaged DNA, the apoptosis induced by chemotherapeutic agents fails. The nucleotide excision DNA repair cross-complementation group 2 *ERCC2* non-synonymous Lys751Gln SNP *2251A*>*C* (rs13181) has still not been recognized as taking part in the mechanism of taxane toxicity/resistance. Our data confirm the lack of correlation as previously described by other authors (Kus et al., 2016).

The *XRCC3* gene (chromosome 14q32) encodes a component of the RecA/Rad51-related protein group. It is a DNA repair protein with an active role in preserving chromosome stability and repairing DNA double-strand breaks. Its reduced activity is associated significantly with SNP Thr241Met *316A*>*G* rs1799794 either through AA or AG alleles. An uncommon SNP in *XRCC3* is related to cancer in patients of altering radiosensitivity (Zou et al., 2014).

In particular, it has been reported in a meta-analysis that *XRCC3 316A*>*G* Thr241Met (rs1799794) is related with response to platinating agents, which highlights the prognostic value of *XRCC3* Thr241Met polymorphism in patients with lung cancer. A meta-analysis of a total of 14 appropriate studies including a total of 2828 patients treated with platinum drugs showed that subjects with the variant 241Met phenotype resulted statistically significant (good outcome) in comparison to those carrying the wild-type 241Thr phenotype (Met vs. Thr, OR = 1.453, 95% CI: 1.116–1.892, *p* = 0.968 and Thr/Met+Met/Met vs. Thr/Thr, OR = 1.476, 95% CI: 1.087–2.004, *P* = 0.696). This noteworthy connection was identified in the Caucasian but not in the Asian population (Qiu et al., 2013). The functional effect of these variants on taxane molecules is unknown, and to date, no study on clinical trials including taxanes has been published. In our hands, *XRCC3 316AA* and *AG* alleles yielded statistically significant results: the OR for all neutropenia grade was 1.52 (95% CI: 0.51–4.91) for GA alleles, and the OR for GG+GA was 2.61 (95% CI: 0.91–7.61) *P* = 0.03.

**Figure 1:**
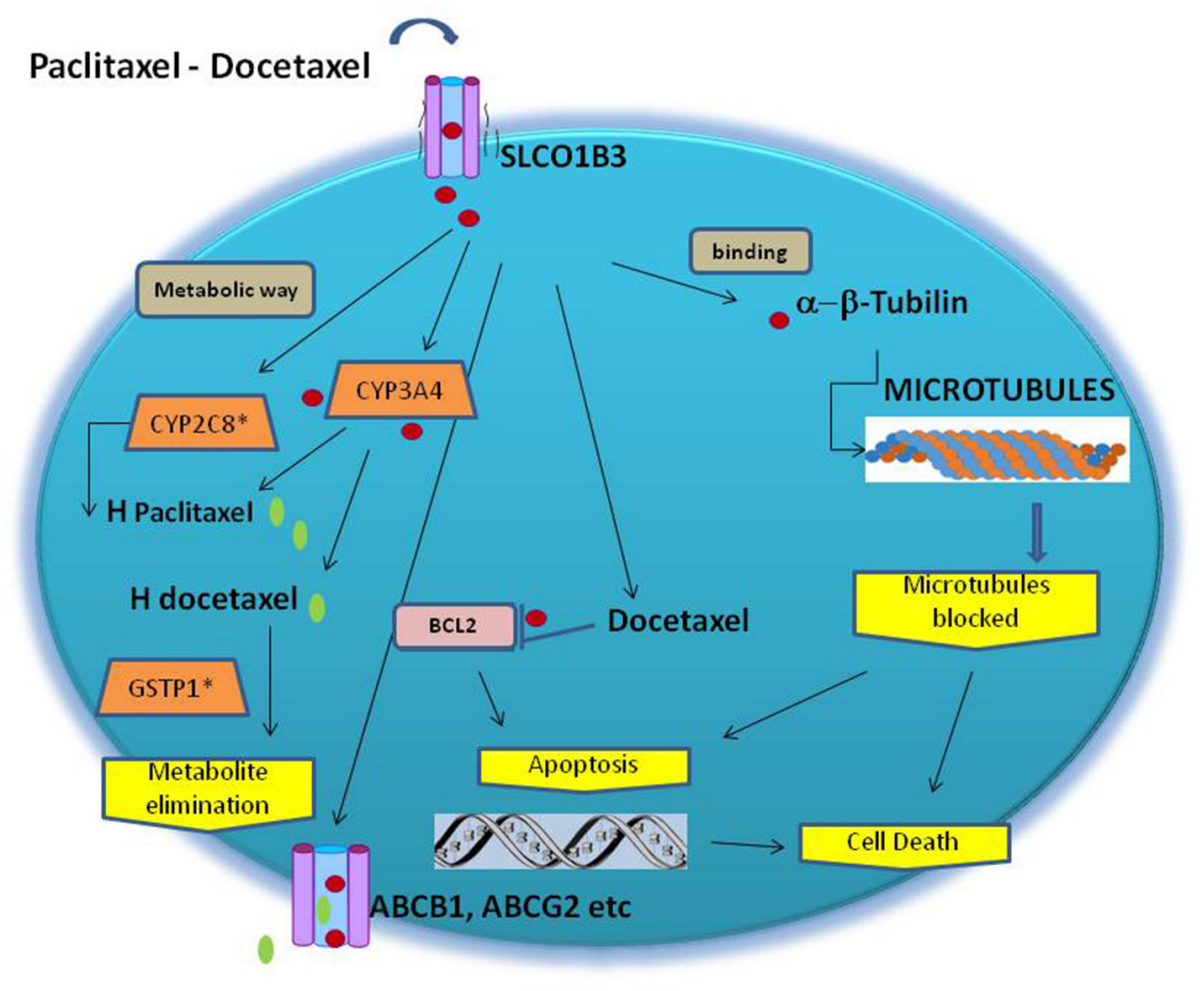
Schematic of the genes and their polymorphism involved in taxane toxicity. Microtubules are composed of β-tubulin and α-tubulin heterodimers. Taxanes block cell division by binding to α-tubulin in the structured microtublules, stabilizing the microtubules, leading to cell death. H-paclitaxel and H-docetaxel are hydroxylated metabolite. Both paclitaxel and docetaxel are extruded by the ATP binding cassette multidrug transporters ABCB1, ABCG2, ABCC1 and ABCC2. CYP2C8 and CYP3A4 are the primary metabolic routes.

Additional gene variants influencing the pharmacodynamics of taxane have been documented. They included Beta-tubulin 2A (TUBB2A) and the role of the polymorphisms rs909964 and rs909965 detected by GWAS (Figure 1). These variants need more evidence in confirmatory studies. In addition, it was associated with pharmacokinetic outcomes but not in neuropathy/neurotoxicity (Abraham et al., 2014).

### Conclusion and future outlook

The clinical effectiveness of the polymorphism described here, could help in developing new diagnostic tool for driving treatment decisions (Frederiks et al., 2015). In particular, molecular testing for a mutation in the *ABCB1* (*alias MDR1*), CYP3A4^*^1B, CYP2C8^*^3, and *XRCC3* genes will possibly help oncologists to select subjects who are most expected to avoid taxane neurotoxicity. For assessing a basic profile of patients responding well/poorly, a panel test of five genetic variants is planned (Table 3).

The aspects addressed here could help clinicians to stratify patients' profiles from genotype A (the most likely responders to treatment) to genotype C (bad responders with higher odds risk of acute and cumulative neurotoxicity). The PGx profile defined as low risk for toxicity showed wild-type expression of *ABCB1* (*3435CC* and *2677GG*), conferring the normal intrusion/extrusion of taxane and active metabolites. In addition, the regular *CYP2C8* and *CYP3A4 392GG* allotypes ensure appropriate metabolic activity. Also, *XRCC3 316AA* exhibits regular expression, as well as normal DNA repair activity.

Pharmacogenomic profiles can show a predisposition to a higher neutropenia and neuropathy risk by reveling higher transmembrane expression of the ABCB1 (3435TT and 2677TT/AA), variant phenotype, conferring the excessive extrusion of taxane from neoplastic cells and causing high plasma concentration. Also poor metabolic activity due to CYP2C8^*^3 (399R) and CYP3A4^*^1B (*392 AA/AG*) causes pharmacokinetic problems, and lower expression of the *XRCC3 316AG/GG* phenotype probably interferes with DNA replication of neoplastic cells and less likely with that of hematopoietic cells, resulting in severe neutropenia, as previously observed in a Caucasian population (Qiu et al., 2013).

There have been certain restrictions in our projected panel tests: (i) these PGx signatures need to be validated in multiple clinical trials with a larger number of patients; (ii) our genotyping data are limited to a Caucasian population; (iii) we did not adjust our data for multiple comparisons (i.e., type of cancer) due to a low number of cohort samples; (iv) the selection of the gene variants was made on the basis of recent findings in clinical trials with significant correlations between the PGx profile and taxane treatments. However, with regard to the gene variants analyzed in this pilot study, the single endpoint was to evaluate the usefulness and cost-effectiveness of a PGx panel assay suitable for application in clinical practice, with particular attention to so-called “frail patients” (Berretta et al., 2013a, 2016).

Furthermore, defining an individual PGx profile does not afford a unique target to assess the optimal strategic approach for the management of taxane-induced neuropathy; thus, it is necessary to seek complementary and alternative medicines (Berretta et al., 2017), as well as to look at nutrition (Berretta et al., 2013b).

In the next few years, it can be expected that there will be links between pharmaceutical and biotechnology companies to undertake larger and broader studies validating tests available for routine diagnostics in pharmacogenomics concerning paclitaxel and docetaxel. Currently, our proposed pharmacogenomic panel assay is useful because it is low cost (about €100,00/genotype/patient) and it is suitable for most clinical laboratory with real time-PCR equipment. In addition, high genomic expertise is not needed to interpret genotype results (Table 3).

In summary, clinicians and laboratory managers should join in evaluating the benefits and limitations, particularly regarding costs and applicability, of the pharmacogenomic tests that are likely suitable for routine clinical practice integration.

## Author contributions

RD and MB study designed and wrote the draft. LA, CF, and TM recruited patients and samples. SD analayzed the samples. SR and GF made data extraction. AC analyzed statistically the results.

### Conflict of interest statement

The authors declare that the research was conducted in the absence of any commercial or financial relationships that could be construed as a potential conflict of interest.
