# Supplementary material for: Assessment of Pharmacogenomic Panel Assay for Prediction of Taxane Toxicities: Preliminary Results
Source: Front Pharmacol. 2017 Nov 7;8:797. doi: 10.3389/fphar.2017.00797 (PMC5682021; doi:10.3389/fphar.2017.00797)
Supplement: Supplementary file 1 [file Table1.docx]

Supplementary Table

# Supplementary Material

**Supplementary table 1**: primers and probes sequences

| **name** | **Primers 5’-3’** | **Probes 5’-3’ mgb** (*minor groove binding*)§ |
| --- | --- | --- |
| CYP2C8*3  rs1050981 | Fw TGGCCAGGGTCAAAGATATTTG  Rev GCATTACTGACTTCCGTGCTACA | Fam TTAGGAAATTCT**C**TGTCATC  Vic TTAGGAAATTCT**T**TGTCATC |
| #CYP3A4*22  rs35599367 | Fw TAGGTCTAATTCAGTTCAGTGTCTCCAT  Rev TTATCAGGTGCCAGTGATGCA | Fam CACCCAG**C**GTAGGG  Vic CCCAG**T**GTAGGGCC |
| GSTP1  rs1695 | Fw GGCAGGGTCATAGATAATTG  Rev CACTACTCGACTACGTGCTCCA | Fam CAAATAC**G**TCTCCCTCA  Vic CGTCAAATAC**A**TCTC |
| ERCC2  rs13181 | Fw ACCAGGGCCAGGCAAGAC  Rev CAGATTGCTCAGCAGCTCTGA | Fam CAGGAGTCACCA**G**GAA  Vic CAGGAGTCACCA**T**GAA |
| *SLCO1B1*  rs4149056 | Fw AAGGAATCTGGGTCATACATGTG  Rev CCCCTATTCCACGAAGCAT | Fam ATATATG**T**GTTCATGGGTAA  Vic ATATATG**C**GTTCATGGGT |
| *ABCG2*  rs2231137 | Fw CAGTTTTACTCGATAGCAGATGTTTATGA  Rev TGTCTTCCAGTAATGTCGAAGTTTTT | Fam TTCCTTGTGACA**T**TGG  Vic TTTCCTTGTGACA**C**TGG |
| *XRCC3*  rs1799794 | Fw TCCACTGACGGATAACAGACTCA  Rev AGCCAGGCCTGTTAAACCAA | Fam CCCTCTGTGCACA**C**CCTGCTGAG  Vic CCCTCTGTGCACA**T**CCTGCTGAG |

§ the single nucleotide polymorphism variant were underscored in bold

# design of the primers and probes were made on complementary DNA strand (A>G)
