# Supplementary material for: Assessment of Pharmacogenomic Panel Assay for Prediction of Taxane Toxicities: Preliminary Results
Source: Front Pharmacol. 2017 Nov 7;8:797. doi: 10.3389/fphar.2017.00797 (PMC5682021; doi:10.3389/fphar.2017.00797)
Supplement: Supplementary file 2 [file Table2.docx]

**Supplementary Table 2.** Distribution of genetic polymorphism according to neutropenia and neurotoxicity, with the distinction for grading among Taxane users only (n=35)

|  | **No adverse events**  **(N=12)** | **Neutropenia & Neurotoxity**  **G1, G2**  **(N= 18)** | **Neutropenia & Neurotoxity**  **G3, G4**  **(N=5)** | **p-value^*^** |
| --- | --- | --- | --- | --- |
| **ABCB1 Iso1145Iso** |  |  |  | 0.20 |
| -Low risk “CC” | 6 (50.0) | 9 (50.0) | 3 (60.0) |  |
| -Middle risk “CT” | 5 (41.7) | 5 (27.8) | 1 (20.0) |  |
| -High risk “TT” | 1 (8.3) | 4 (22.2) | 1 (20.0) |  |
| **ABCB1 Ala893Ser** |  |  |  | 0.90 |
| Low risk “GG” | 7 (58.3) | 8 (44.4) | 3 (60) |  |
| Middle risk “GC” | 4 (33.3) | 9 (50.0) | 2 (40) |  |
| High risk “CC” | 1 (8.4) | 1 (5.6) | 0 |  |
| **CYP3A4*1B5 UTR** |  |  |  | 0.30 |
| Low risk “AA” | 6 (50.0) | 8 (44.5) | 4 (80.0) |  |
| High risk “AG+GG” | 6 (50.0) | 10 (55.5) | 1 (20.0) |  |
| **CYP2C8*3** |  |  |  | 0.70 |
| Low risk “TT” | 8 (66.7) | 11 (61.1) | 4 (80.0) |  |
| Middle risk “CT” | 4 (33.3) | 7 (38.9) | 1 (20.0) |  |
| **CYP3A4*22** |  |  |  | 0.60 |
| Low risk “CC” | 10 (83.3) | 15 (83.3) | 5 (100) |  |
| Middle risk “CT” | 2 (16.7) | 3 (16.7) | 0 |  |
| **GSTP1** |  |  |  | 0.06 |
| Low risk “AA” | 4 (33.3) | 6 (50) | 5 (100) |  |
| Middle risk “AG” | 8 (66.7) | 6 (50) | 0 |  |
| **ERCC2** |  |  |  | 0.30 |
| Low risk “TT” | 4 (33.3) | 9 (50) | 1 (20) |  |
| Middle risk “GT” | 4 (33.3) | 8 (44.4) | 3 (60) |  |
| High risk “GG” | 4 (33.3) | 1 (5.6) | 1 (20) |  |
| **SLCO1b1** |  |  |  | 0.40 |
| Low risk “TT” | 5 (55.6) | 12 (80) | 4 (80) |  |
| Middle risk “CT” | 4 (44.4) | 3 (20) | 1 (20) |  |
| **ABCG2** |  |  |  | **0.02** |
| Low risk “CC” | 10 (83.3) | 15 (83.3) | 2 (40) |  |
| Middle risk “CT” | 2 (16.7) | 1 (5.6) | 0 |  |
| High risk “TT” | 0 | 2 (11.1) | 3 (60) |  |
| **XRCC3** |  |  |  | 0.60 |
| Low risk “AA” | 7 (58.3) | 13 (72.2) | 3 (60) |  |
| Middle risk “AG” | 3 (25) | 2 (11.1) | 2 (40) |  |
| High risk “GG” | 2 (16.7) | 3 (16.7) | 0 |  |

In bold is significative result (*P*<0.05)
